# Antioxidants in Animal Nutrition

**DOI:** 10.3390/antiox10121877

**Published:** 2021-11-25

**Authors:** Carlo Corino, Raffaella Rossi

**Affiliations:** Department of Veterinary Medicine, Università degli Studi di Milano, Via Dell’Università 6, 26900 Lodi, Italy; raffaella.rossi@unimi.it

Oxidative stress is an imbalance between the production of free radicals and their neutralization by the antioxidants’ defenses [1]. A low amount of reactive oxygen species (ROS) are required for the physiological functions of the organism; however, an excess causes oxidative damage in several molecules, negatively influencing the cells’ DNA and protein as well as causing the lipid peroxidation of cellular membranes [2]. 

In livestock, there is a well-defined correlation between the onset of some diseases and a reduction in the antioxidant status [3]. Oxidative stress is also implicated in many pathological disorders that impair animal health, welfare, and productive parameters [4]. In fact, in some productive phases, animals have to deal with physiological changes, such as farrowing and lactation, or environmental changes, such as weaning or heat temperature, and several types of stress cause a decline in the antioxidant status [5,6,7]. A good antioxidant status in animals can also positively affect meat quality parameters, improving meat Vitamin E content and decreasing meat lipid peroxidation [8,9]. 

The aim of this Special Issue of *Antioxidants* entitled “Antioxidants in Animal Nutrition” is to publish papers and reviews on the effects of feed antioxidants on animal performance and health and product quality. It comprises five reviews and eleven original research papers.

Firstly, dietary challenge models, including oxidized fats and oils, heavy metal exposure, soybean meal, protein or amino acids, and diets contaminated with mycotoxins are reviewed. These models facilitate our understanding of the physiological mechanisms involved and the most suitable antioxidants that can mitigate oxidative stress in poultry, swine, and fish [10]. 

Consumers tend to focus on safe and natural products of animal origin, and thus it is important to substitute synthetic substances with natural ones. There have been several recent studies on plant feed additives used as natural alternatives to synthetic antioxidants. In fact, natural extracts, essential oils, and by-products from plants contain bioactive compounds that are strong natural antioxidants. The effects of natural extracts on product quality, oxidative stability, and shelf life in ruminants, swine, and rabbits have been reviewed. The authors reported that the effects of plant additives are different but similar to synthetic vitamin E, thus suggesting their role as possible natural substitutes [11]. The impact of plant additives in poultry nutrition has also been analyzed, based on the selection of literature published in the last 20 years [12,13]. Plant additives have been found to be a valuable strategy to reducing oxidative stress in poultry, with positive effects on growth performance. However, it is important to find the right dosage of the various extracts to prevent problems such as feed digestibility and negative effects on gut morphology [12]. A positive influence of dietary plant extracts on oxidative stability, sensory characteristics, and fatty acid composition of poultry meat has also been observed, suggesting the partial substitution of synthetic antioxidants [13].

The effects of heat stress on broiler antioxidant status and inflammatory responses have also been analyzed because such stress is an important problem in the poultry sector. The authors highlighted that some studies have reported that dietary supplementation with extracts of *Salix spp.* bark in heat-stressed broilers reduced oxidative stress markers and enteric pathogenic bacteria, thus improving growth performance [14].

In this Special Issue, four original research papers deal with the study of plant feed additives as natural alternatives to the use of synthetic antioxidants. The first of these four papers reports that dietary supplementation with natural extracts containing polyphenols in gestating sows increased the number of piglets born alive and the litter weight, decreasing the presence of low-birth-weight piglets. In addition, piglets born from sows receiving polyphenols had a higher antioxidant status and tended to have a higher weaning weight than controls [15]. These data underline the importance of antioxidant status on the productive performance of sows and piglets. In the second study, the effects of several plant extracts on antioxidant status and reserves in weaned piglets were evaluated using the Kit Radicaux Libres test (KRL^TM^). To the best of our knowledge, this is the first study to assess the antioxidant reserves in animal nutrition. Antioxidant reserves help identify the amount of antioxidants stored in the organism, which can be released to reduce oxidative stress. *Boswellia* and plant extract mixtures were found to sustain blood antioxidant status, while *Uncaria* and *Tanacetum* extracts are mainly converted in blood reserves in the form of glucuronides, sulphates, and glucosides. It is thus interesting that different phenolic compounds play a different role in the organism, and this should be related to the phenolic metabolism and their relationship with the intestinal microbiome [16]. 

Another study, conducted on broilers exposed to heat stress, showed that dietary supplementation with astaxanthin, a carotenoid derived from *Phaffia rhodozyma* yeast, improved the antioxidant status, immune response, and meat quality [17]. In the fourth study, the dietary inclusion of hazelnut peel in dairy ewes was investigated. This is a by-product from the chocolate industry and is rich in phenolic compounds. The data showed that cheese from hazelnut peel supplemented ewes has a healthier lipid profile and higher tocopherol content [18].

The effects of different feed ingredients, selenium form and rumen-protected amino acids, on antioxidant status in ruminants were evaluated in four experimental trials. In the first one, the administration of fresh or dry forage affected the oxidative stress index in lactating goats. The effects of forage polyphenols on the antioxidant status of lactating goats are not conclusive, and thus further studies are needed [19]. In another trial, dietary sesame meal in goats at the mid-lactation stage was also investigated and was found to be a valuable strategy to reduce oxidative stress markers and increase the milk oxidative stability. Dietary sesame meal in combination with synthetic antioxidants (vitamin E and selenium) had a negative effect on antioxidant status. This study highlighted that the over-consumption of synthetic antioxidants such as vitamin E and selenium, leads to a situation identified as ‘antioxidative stress’, with detrimental effects on the organism [20]. However, a study on hydroxy-selenomethionine, a chemical synthesized organic form of selenium in the diet of Charolaise bulls has shown an improvement in meat quality parameters [21].

Amino acids are involved in the cellular oxidative balance, and the effects of dietary rumen-protected methionine and lysine, alone or in combination, were evaluated in early lactating ewes. The results showed that methionine improved the antioxidant status and, in contrast, lysine reduce milk oxidative stability. The authors reported that attention should be given not only to the dietary amount but also to the amino acid effects in order to prevent physiological imbalance and oxidative stress [22].

In addition, dietary probiotics and postbiotics have been shown to be a good approach to counteract the oxidative stress involved in the onset of several enteric pathologies. This was analyzed in two other original research papers. In the first, dietary supplementation with *Lactobacillus delbrueckii* in piglets, challenged with lipopolysaccharide, improved the intestinal structure and tight junctions and enhanced antioxidant functions, upregulating the nuclear factor erythroid 2-related factor (Nrf2) signaling pathway [23]. In the second one, the antioxidant activity of *Lactobacillus plantarum* postbiotics RG14 in post-weaning lambs was investigated. Dietary postbiotic increased serum and ruminal fluid antioxidant activity and reduced serum lipid peroxidation, and the upregulation of hepatic antioxidant enzymes and ruminal barrier function was also observed [24]. 

An in vitro study analyzed the effects of N-acetyl serotonin, a metabolite of L-tryptophan, on porcine enterocytes submitted to oxidative stress with 4-hydroxy-2-nonenalon. The data showed that N-acetyl serotonin attenuated oxidative stress in porcine enterocytes by regulating Nrf2 signaling. These findings provide new insights into the efficient role of N-acetyl serotonin in preserving the intestinal homeostasis [25].

In conclusion, this Special Issue adds new evidence to the field of the dietary supplementation of antioxidants in livestock, supporting their beneficial role in enhancing antioxidant status and animal health. The product quality was also enhanced by protecting against oxidation and improving their nutritional value. Further studies are needed to better understand the health benefits of dietary antioxidants in livestock and to identify the correct dosage to prevent antioxidative stress.
